# Impact of young people’s admissions to adult mental health wards in England: national qualitative study

**DOI:** 10.1192/bjo.2024.850

**Published:** 2025-03-17

**Authors:** Anne-Marie Burn, Josephine Holland, James Roe, Elinor Hopkin, Lorna Wild, Michelle Fisher, Tamsin Ford, Saeed Nazir, Bernadka Dubicka, Anthony James, Helena Tuomainen, Nicole Fung, Adam Wagner, Richard Morriss, Kapil Sayal

**Affiliations:** Department of Psychiatry, University of Cambridge, Cambridge, UK; School of Medicine, Mental Health and Clinical Neurosciences, University of Nottingham, Nottingham, UK; National Institute for Health and Care Research, Applied Research Collaboration (ARC) East Midlands, University of Nottingham, Nottingham, UK; NIHR Applied Research Collaboration Greater Manchester (NIHR ARC GM), University of Manchester, Manchester, UK; Oxford Health NHS Foundation Trust, Oxford, UK; National Institute for Health and Care Research, Applied Research Collaboration (ARC) West Midlands, Warwick Medical School, Coventry, UK; Nottinghamshire Healthcare NHS Foundation Trust, Nottingham, UK; Hull and York Medical School, University of York, York, UK; School of Health Sciences, University of Manchester, Manchester, UK; NIHR Applied Research Collaboration West Midlands, University of Warwick, Coventry, UK; Birmingham Women’s and Children’s Hospitals NHS Foundation Trust, Birmingham, UK; National Institute for Health and Care Research, Applied Research Collaboration (ARC) East of England, University of East Anglia, Norwich, UK

**Keywords:** In-patient treatment, qualitative research, child and adolescent psychiatry, mental health services, patients

## Abstract

**Background:**

National policy in England recommends that young people be admitted to mental health wards that are age-appropriate. Despite this, young people continue to be admitted to adult wards.

**Aims:**

To explore the impact of young people’s admissions to adult wards, from the perspectives of young people, parents/carers and mental health professionals working in adult services.

**Method:**

Semi-structured interviews were conducted with 29 participants to explore experiences of receiving and delivering care in adult mental health wards. Participants were four young people (aged 16–17 years), four parents/carers and 21 mental health professionals from adult mental health services in England. Data were analysed using framework analysis.

**Results:**

Young people’s admissions to adult wards tend to occur out of hours, at a time of crisis and when no suitable adolescent bed is available. Admissions were conceptualised as a short-term safety measure rather than for any therapeutic input. Concerns were raised about safeguarding, limited treatment options and a lack of education provision for young people on adult wards. However, exceptionally, for older adolescents, an adult ward might be clinically or socially appropriate. Recommendations to reduce adult ward admissions included better integration of adolescent and adult services, having more flexible policies and increasing community provision.

**Conclusions:**

Our findings emphasise the importance of young people being admitted to age-appropriate in-patient facilities. Earlier intervention and increased provision of specialist care in the community could prevent young people’s admissions to adult wards.

An increasing number of children and young people in the UK are suffering from mental health disorders, with many needing referral to, and input from, child and adolescent mental health services (CAMHS).^1,2^ Since the COVID-19 pandemic, there has been a steep rise in the number of young people aged <18 years who need crisis care, which might reflect an increased severity in presentations.^3–5^ An in-patient admission may be required for those who are at high risk and/or have complex mental health needs that cannot be safely met within the community. However, there is considerable geographical variation in adolescent in-patient provision across the UK, with some areas having limited access to beds locally.^6,7^ Consequently, many general adolescent wards are at capacity and unable to admit at short notice.^7,8^ The increasing demand for services combined with a lack of capacity can create difficulties in identifying adolescent beds, particularly on an urgent basis, often resulting in young people being admitted to acute paediatric wards, adolescent wards far from their homes or adult mental health facilities.^8–12^

National policy and legislation specify that young people should be cared for in an in-patient facility appropriate for their age.^13,14^ Despite this, adolescents are still sometimes admitted to adult wards,^15^ including children as young as 12 years.^16^ Admissions of this type usually occur in an emergency when no suitable adolescent bed is available, and cases often include young people detained by the police under Section 136 of the Mental Health Act 1983 (MHA) who are then admitted to an adult ward.^15,17^ Studies have identified a number of adverse experiences for young people on adult wards, raising concerns about their comfort, treatment and safety. For instance, young people have reported feeling isolated and bored on adult wards because of the lack of age-appropriate activities and schooling.^18–20^ Adult clinical teams often lack paediatric mental health training and therefore may not have the skills and expertise to manage adolescent patients, and consequently, young people may not receive age-appropriate care.^9,18^ Clinical staff reported that young people often feel frightened and unsafe around adult patients, and may witness distressing events such as self-harm and suicide. Staff expressed concerns about young people being at risk of harm, exploitation or even learning maladaptive behaviours from adult patients.^16,18^ Such negative healthcare experiences may deter young people from seeking help or engaging with services, and lead to poor long-term health and educational outcomes.^20^

## Aims

Little is known about the impact of young people’s admissions to adult psychiatric facilities.^21^ The aim of this study is to explore the views and experiences of young people, parents/carers and mental health professionals (MHPs) in adult mental health services (AMHS), regarding the treatment and care of young people under 18 years of age in adult mental health units, and to identify ways to improve services for this population.

## Method

### Study design and setting

This qualitative research was part of the ‘Far Away from Home’ research programme and the results from associated studies are reported elsewhere.^17,22^ We conducted in-depth semi-structured interviews with young people, parents and MHPs working in the English National Health Service (NHS). Participants were identified and recruited from community and secondary care mental health in-patient settings in England. Reporting followed the Consolidated Criteria for Reporting Qualitative Studies (COREQ) guidelines (Supplementary File 1).

### Participant recruitment

Young people and parents were recruited through local participating NHS Trusts in three large regions in England (East Midlands, East of England and Greater Manchester) between February 2021 and September 2022 (19 months). We developed a predetermined sampling frame to purposively recruit young people (aged 13–17 years) and parents/carers with experience of being admitted to an adult mental health ward. As far as possible, we aimed to recruit a diverse sample of young people across the age range, gender and ethnicity. Potentially eligible participants were initially approached by a clinician who was part of the young person’s usual care team. Clinicians explained the study and provided young people and parents with the information sheet. Participants who expressed an interest provided their details on a ‘declaration of interest’ form, which was then passed to the study team.

We recruited MHPs in AMHS with experience of caring for young people aged <18 years via a national surveillance survey,^17^ and using a snowball approach through four local participating sites (East Midlands, East of England, Greater Manchester and Oxford and Thames Valley regions). We sought perspectives from a diverse range of health professionals, including in-patient consultant psychiatrists, nurses/team leads, ward managers and service commissioners.

### Procedures

We developed interview schedules for each stakeholder group in collaboration with the study’s professional advisory group and the parent/carer Lived Experience Advisory Panel (Supplementary Files 2–5). The interview gathered participants’ experiences and perceptions regarding admissions, the impacts on the treatment and care of patients (including benefits and harms), and suggestions for improvement of service organisation and delivery.

Information sheets outlined the aims of the study, including details about participants’ confidentiality and data storage, and contact details for the research team. Written informed consent was obtained from all participants before the interviews, and ethical procedures for secure data storage were followed. Interviews were conducted by a team of experienced qualitative researchers (A-M.B., E.H., J.H., J.R., L.W. and M.F.), and took place remotely over video call or telephone, or in-person in secondary care in-patient settings. Interviews lasted between 25 and 60 mins and were audio-recorded, transcribed verbatim and de-identified to protect the confidentiality of the participants. Young people and parents were given an online voucher (£15) in appreciation for their time.

### Analysis

Interviews were thematically analysed using the Framework Method,^23,24^ a flexible and pragmatic approach that enables the comparison of data from different stakeholder groups. It is particularly useful for projects involving a large multidisciplinary team, and facilitates the involvement of lay members in the analytic process. Analysis followed five stages: familiarisation with the data, identifying a framework, indexing, charting and mapping, and interpretation. Six members of the research team (A-M.B., E.H., J.H., J.R., L.W. and M.F.) began the analysis by reading and deductively coding a selection of overlapping transcripts to identify *a priori* codes based on the research questions and topic guide, and inductively developed emergent codes. Through a whole-group discussion, we developed three working analytical frameworks, one for each stakeholder group, including a brief summary to describe each subtheme. The research team met with the professional advisory group and parent/carer Lived Experience Advisory Panel to discuss the interview data and involve them in the process of analysis. Some advisory group members read excerpts from the transcripts and provided valuable insights during the coding phase. The working analytical frameworks were entered into NVivo, version 12 for Windows (Lumivero LLC, Denver, Colorado, USA; https://help-nv.qsrinternational.com/12/win/v12.1.115-d3ea61/Content/welcome.htm), and applied to the remaining transcripts. The team met regularly to discuss the coding and refine the frameworks so that they encompassed all data. When coding was completed, we exported the framework matrices into Microsoft Excel (Microsoft Office 365 for Windows) and began charting the data by each respondent, which involved producing concise summaries of the verbatim text in each matrix cell. We regularly checked the charting to ensure that we were summarising the data consistently across the team members. In the final interpretation stage of the analysis, summaries of each subtheme were developed, which enabled the team to explore patterns within the data and across stakeholder groups to identify overarching themes.

### Ethical approval

The authors assert that all procedures contributing to this work comply with the ethical standards of the relevant national and institutional committees on human experimentation and with the Helsinki Declaration of 1975, as revised in 2013. All procedures involving human patients were approved by West Midlands – South Birmingham Research Ethics Committee (approval number 20/WM/0314).

## Results

Twenty-nine people participated in the study. Twenty-one MHPs from AMHS across all NHS regions in England were interviewed, including adult consultant psychiatrists (*n* = 10), nurses/team leads (*n* = 5), ward managers (*n* = 2) and service commissioners (*n* = 4). Recruitment of young people and parents was challenging and resulted in a smaller sample size than expected; further details are provided in the ‘Study limitations’ section. Four young people aged 16–17 years (three females and one male) and four parents/carers (three females and one male) were recruited from three regions in England: East of England, East Midlands and Greater Manchester. At the time of admission, young people were 16–17 years of age (median 17 years).

Four main themes were generated from the data and are summarised in Table 1. Example quotes are referred to in the text from a range of participants and given in full in Tables 2–5.

**Table 1:** Main themes and subthemes

Theme 1	Theme 2	Theme 3	Theme 4
Admissions to adult wards are the ‘last resort’	Appropriateness of adult wards for young people	Impact on services and provision of care	Recommendations for service provision and policy
• Lack of adolescent beds in a crisis Challenges of involving families in the referral process Admissions are intended as a short-term safety measure	• Concerns about patients’ comfort, risks and safeguarding Lack of education provision and age-appropriate activities Wards may be clinically or socially appropriate for a small subset of young people	• Level of care for young people on adult wards Some safeguarding protocols are felt to be intrusive and restrictive Barriers and facilitators to collaborative working	• Integration of services Increase community provision Recommendations for policy

**Table 2:** Quotes illustrating theme 1

Quote	Admissions to adult wards are the ‘last resort’
1	…the emergency pathways from whichever area, don’t really take effect and the local support within the out-of-hours support isn’t really there. So, the least harm placement at that time is deemed to be the adult ward, for whatever reason. (MHP)
2	…it happens very, very rarely. The occasions that I am aware of is when the young person is due to be turning 18 within the next few weeks or possibly a month or two. (MHP)
3	…when I do on-calls as an adult consultant, that we see a young patient detained under Section 136. And again, whilst before I had never seen an under-18 in a psychiatric ward, now we have five a year and that’s probably common across all the wards. So, it used to be a very rare event and now it’s not that rare an event. (MHP)
4	She took a big overdose and disappeared and we had police out and drones and things looking for her… and she was then taken to [Hospital 1] and then there was a big discussion about where she would go. They said there’s no adolescent beds. (Parent)
5	…they had to find me somewhere to go as soon as possible and there was beds available at [unit name], so that’s why I went to an adult ward. (Young person)
6	…they [parents] just wanted her to be in a place of safety really. (MHP)
7	It was said that this was an adult ward, I think it was explained that this would be a shorter-term solution and actually we’re looking at other alternatives. I just don’t think they [the parents] fully understood prior to arriving on the ward what that meant. Where actually, when you get here and you see the ward and you meet the other service users, I’m not sure it always gives them the same reassurance that it had done when they were in A&E [Accident and Emergency]. (MHP)
8	It made me feel like other people were controlling my life and I wasn’t involved. (Young person)
9	They literally said the ambulance is going to be here in about half an hour to come pick you up, you’re going to [adult ward name]. And I was like pleading not to go, I really didn’t want to go but I had to. (Young person)
10	They didn’t even tell me I was going on an adult ward. They was like oh you’re being admitted to [Hospital Name] because there’s no CAMHS beds. So, I thought oh it’s just the general but then I ended up on [adult ward]. (Young person)
11	…I think he [patient] was so distressed, I mean I don’t think he would have been able to even take on fully what was going on. (MHP)
12	…even if I take them, this is like a short-term measure, once a patient is stabilised or whatever and then once the bed comes locally, we can repatriate. Doesn’t mean that they have to stay here forever. (MHP)
13	So, I was only meant to be there for a few days and I was there 6 weeks… for the first 3 or 4 weeks I was waking up every day being like oh I hope today’s the day that I can go to one that’s more suitable for me and my needs. (Young person)
14	I just kept thinking it would be a couple of days before a CAMHS bed would be available, but days turned into a week, a week turned into 2–3 weeks. (Parent)

MHP, mental health professional; CAMHS, child and adolescent mental health services.

**Table 3:** Quotes illustrating theme 2

Quote	Appropriateness of adult wards for young people
1	Wards are very loud, scary and intimidating for young people. (MHP)
2	I think they found it quite distressing. I think initially very scared of the environment, not wanting to come out of the bedroom, not wanting to spend time on the ward, isolating themselves in that area. I can imagine if I was that age and being with a group of adults but adults with mental health issues as well, that must be terrifying. (MHP)
3	…if they are like between 13 and 16, I wouldn’t be very happy. (MHP)
4	…if you think about a 14-year-old and then like a 21-year-old maybe with some personality disorder you know, who’s talking to a 14-year-old, I don’t know, I just feel … you know how easily-influenced you know young people are anyway. (MHP)
5	They can learn behaviours with each other there… “Oh, you started cutting yourself, what does it do for you? I can start too”, even if they had not before. (MHP)
6	…[daughter] mentioned an incident once where a young lad had got socks and tried to hang himself. You know, it’s a lot isn’t it? It’s a lot for your child to have to witness. And then I worry is anyone going to say things to her or put ideas into her head, you know – “you self-harm, I’ll self-harm”. (Parent)
7	I didn’t feel comfortable going out into communal areas because I didn’t feel comfortable being with older people. (Young person)
8	Like it affected me in so many different ways I can’t even explain, like it brought back so much of my flashbacks, so much of my PTSD [post-traumatic stress disorder] and like it just made me ten times worse. (Young person)
9	I understand why there’s no school because it is for adults and they’re not part of school but there is no replacement for that during the day. (Young person)
10	…when I first came there was nothing, there was no groups, no … like obviously no school but like nothing was happening in the day. And so I kept myself entertained in my room but even if there was things on, I don’t think I would have wanted to join in just because of … I didn’t feel comfortable with a big range of ages. (Young person)
11	…partly because of the other residents were older but also the turnover was very high. So, there was nobody that she could form a bond with because that person would be gone in 2 or 3 days’ time. (MHP)
12	We tried to engage with her with games and stuff, but when you’ve got other people coming in and there’s only two staff on the ward, that takes precedence. So, she would have been ignored to some degree, not through conscious effort. (MHP)
13	I think again it has to be factored in around risk and the maturity and what their need is. (MHP)
14	…if this person’s living independently and got a full-time job, rides a moped, smokes cigarettes, wants their mobile phone, a CAMHS in-patient ward is probably not suitable. (MHP)
15	…for some of them, they would have posed quite a bit of risk to younger people. (MHP)

MHP, mental health professional; CAMHS, child and adolescent mental health services.

**Table 4:** Quotes illustrating theme 3

Quote	Impact on services and provision of care
1	We had a very, very young patient, 15-year-old, she was a very young 15, that I really felt out of my depth in just talking to her. Even I have children of that age but obviously it’s a very different environment where we struggled to relate to her clinically. (MHP)
2	…her actual functioning age was more around a child of 8 or 9 years old, it was a very different nursing style, which I think we don’t necessarily have experience or training in, and so I think it’s then quite difficult to manage at times, to support the staff to manage that. People were definitely uncomfortable. (MHP)
3	What becomes a bit more difficult is the philosophy or the ethics of the treatment. And so, we would be aiming at a quick discharge. So, it will take a few days to manage a crisis, 3–5 days, and then we will discharge them, even if the crisis continues to be present. Because there is not much more that will change by them remaining in hospital. The CAMHS perspective is very different. So, they don’t seem to worry too much about admitting a patient and the patient just staying for months or much longer, with no clear planned therapy or purpose for the patient to remain in hospital. (MHP)
4	…we did not medicate the patient; we didn’t overreact to her ideas of self-harm. (MHP)
5	The staff didn’t really know how to handle … and they said this to us, they weren’t used to having adolescents. They didn’t know how to handle her really. (Parent)
6	She didn’t have treatment, all that was done was keeping her in her room and making sure she took medication with occasional escorted walks until she ran away from one. A couple of times they did that and she ran away while she was on an escorted walk, so then they stopped doing that. (Parent)
7	…in (CAMHS Unit) he gets loads of different interventions, occupational therapy, education, psychological, all of that. He didn’t get any of that there [adult mental health ward], and also, they weren’t tending to his personal needs. (Parent)
8	So that was basically someone just sitting at my doorway. Making sure I’m safe. But in terms of like therapy and stuff, the only support I was offered was an OT [occupational therapist], who I did meet with weekly and she would be the one person I did something with in the week. And I got on really well with her but in terms of like psychology or therapy I received like none of that. (Young person)
9	I felt like they were just letting me down again because obviously I feel like you don’t get as much help on an adult ward as you do when … so Adult Services isn’t as good as CAMHS Services, like Children’s Services because you get the psychology input and the doctor’s input and stuff like that. Whereas if you’re on an adult ward you’re basically just left to sort yourself out and then get discharged. (Young person)
10	…[the] patient’s family felt that she was imprisoned in our unit. (MHP)
11	We explain what we are doing and why we are doing it, so they understand that it’s not because of what they’ve done or what they will do, it’s just policy. (MHP)
12	…they [CAMHS consultant] are involved in the reviews and the care plans and everything that needs to happen to this young person. (MHP)
13	It would just be nice to do a bit more joined-up working but that requires resource and we don’t have that resource. (MHP)
14	I think the difficulty we have, the fundamental problem we have is around Social Care, whether it be local or distant. That is what stalls our discharge. (MHP)
15	I don’t think it’s enough to say there should be good working practices between Social Care, I think we should be mandated to do it you know. If someone is requiring admission to hospital it should be mandatory for Social Care to do an assessment with us together on what this young person’s needs are and the family’s needs are. (MHP)

MHP, mental health professional; CAMHS, child and adolescent mental health services.

**Table 5:** Quotes illustrating theme 4

Quote	Recommendations for service provision and policy
1	If there was a better provision for those hybrids that are not children anymore but they’re not adults either. They’re that funny in-between age and they have really quite specific needs and no service seems designed to meet that. (MHP)
2	It would be nice to have like I say a session a week from a CAMHS psychiatrist that we could say right, we’ve got these few patients we want to discuss with you, as much for kind of supervision and guidance as anything else. (MHP)
3	And we’re caught up in the middle of all this, these arguments between Health and Social Care. There’s no joined-up thinking between the two. And you know, our daughter needs Mental Health Services and she needs Social Care but you know, it’s almost like it’s a triangle and she’s caught in the middle between Health and Social Care. There’s nothing joined-up and there’s no-one fighting on her behalf. (Parent)
4	I think really the solution probably lies with the community and that’s the national direction isn’t it, is enhancing community services and keeping young people in the community as much as possible, which I think is the right way to go. (MHP)
5	Because I feel like sometimes there’s people on children’s wards that don’t actually need to be here anymore and they could get that support in the community. (Young person)
6	All the psychiatrists keep saying she’s better off in the community, she’s got to be in the community, that’s the best way to treat the illnesses that she’s got. Which is great and I agree with that but the support is not available in the community you know. (Parent)
7	We should not be using in-patient services as the default, which sadly it becomes for a lot of young people because they’ve not had the right care and support in the community. And that’s where the investment’s got to be. (MHP)
8	I think if we get early access to care, but this is the current situation at the moment. Someone gets referred in and they’re waiting 2 or 3 weeks, if not months, before they get someone to come and talk to them. (MHP)
9	I think there are certainly a cohort of young people where an admission isn’t in their best interests and that if we had stronger home treatment, stronger and additional capacity and home treatment models that provided support to the family unit as well, I think a lot of that would prevent admissions. (MHP)
10	We’ve seen the enhanced eating disorder services work really, really well and close with young people to prevent them coming into hospital. But again because of how commissioning pathways are, separating of services currently, that isn’t equitable currently, although should be as we move forward into the future. (MHP)
11	It’s about developing and skilling up practitioners and much more system working together from my perspective. (MHP)
12	You know, we have our home-based treatment team and we have [Name of Region] Assessment Centre and they do work weekends but they work ‘til I think it’s 8 or 9 o’clock on a weekend…and we know young people, a lot of crises happen after bedtime, when they’re trying to get to sleep and it’s dark and they’re alone. (MHP)
13	I’m not a CAMHS consultant, I’m a general adult consultant, but I don’t know all the policies, all the regulations, what do you do with the young adults, so I need someone who could be the responsible clinician, I’m happy to assist them as a ward doctor, but I couldn’t be the responsible clinician. (MHP)
14	…levels of observation, especially high levels of observation and intrusive observation, should be measured against risk, not measured against chronological age. (MHP)
15	I think they need to help with the transition to Adult Services. Like I say I’ve made quite a lot of friends and they’re around my age and their transition to Adult Services has been really bad. I think that there’s a big age gap in the adult wards and I think there needs to be a bridge between that. I feel like there should be a unit for later teens/early 20s because you’re still developing at that age…yes you’re classed as an adult but you’re still like getting the independence and developing things. (Young person)

MHP, mental health professional; CAMHS, child and adolescent mental health services.

### Theme 1: admissions to adult wards are the ‘last resort’

#### Lack of adolescent beds in a crisis

MHPs described adult ward admissions as a ‘last resort’ for young people who are ‘acutely unwell, detained and needing treatment’. These admissions typically arise in emergencies when the risks are too high for the young person to be safely managed in the community and a local adolescent bed is not available. Reports suggested that adolescent wards might decline referrals if the patient is older (i.e. 17 years old), with a history of trauma, emotional disturbance and challenging behaviour. Referrals may also be declined when bed capacity is reached or restricted because of staffing issues, or complexities around the care needs of the patient. Adult ward admissions mostly come via the MHA Section 136 route, involving young people at high risk of harm or suicide, and detained for their own safety. These types of admissions are more likely to occur during holiday periods or out of hours, when local provisions are at reduced capacity (Quote 1, Table 2). For some MHPs, adult admissions are a rare occurrence, whereas others had seen a notable increase in cases, thus highlighting a regional variation in experience (Quotes 2 and 3, Table 2).

Parents and young people provided narratives detailing a period of escalating risk preceding the admission, involving multiple suicide attempts, and an adult ward admission was agreed because of a lack of available adolescent beds (Quote 4, Table 2). Before admission, young people had experienced prolonged stays in a general hospital and transfers to Section 136 suites – a special provision for patients detained under the MHA, a situation that they perceived had increased the urgency to admit them to any available facility (Quote 5, Table 2).

#### Challenges of involving families in the referral process

At the time of admission, parents described feeling distressed, struggling to cope and very worried about their child because of a high risk of suicide and self-harm. MHPs spoke about parents being relieved when a bed became available, even an adult bed, because it was seen as a place of safety (Quote 6, Table 2). However, a professional highlighted that parents may not be fully aware about the nature of adult wards before admission, and their perceptions change when they actually visit the ward and interact with the other patients (Quote 7, Table 2).

Young people spoke about a lack of agency in the decision to be admitted to an adult ward, and having insufficient information and limited time to contemplate the situation (Quotes 8 and 9, Table 2). One young person explained that they were not informed that they were being moved to an adult ward, which resulted in distress on arrival (Quote 10, Table 2). Professionals explained that it can be a challenge to involve young people in the decision-making process, because they often lack the capacity at this point to actively engage in decisions about their care (Quote 11, Table 2).

#### Admissions are intended as a short-term safety measure

MHPs explained that admissions are typically short term, describing adult wards as a temporary ‘holding bay’, biding time until a suitable adolescent unit bed becomes available (Quote 12, Table 2). Consequently, adult clinical staff saw their role as managing the patient’s short-term safety while the search for an adolescent bed was ongoing. Some articulated that adult wards are expected to just ‘keep them safe’, rather than providing any therapeutic input for the young person. Despite being told that the admission would be for just a few days, parents and young people experienced longer stays, with one young person remaining on the adult ward for 6 weeks (Quotes 13 and 14, Table 2).

### Theme 2: appropriateness of adult wards for young people

#### Concerns about patients’ comfort, risks and safeguarding

MHPs expressed the view that, in general, young people’s admissions to adult wards are ‘inappropriate’ and should be circumvented. In their experience, young people often feel frightened and unsafe in the presence of adult patients who are severely unwell and exhibit unpredictable behaviour (Quotes 1 and 2, Table 3). They voiced specific concerns regarding the risk and vulnerability of children aged <16 years on adult wards, particularly regarding how to ensure safeguarding and keep children safe around adult patients (Quotes 3 and 4, Table 3). They emphasised the potential risks associated with young people interacting with adult patients who may have significant drug or forensic histories, highlighting the susceptibility of young people forming inappropriate relationships with adult patients. For instance, a young person may exchange telephone numbers with an adult on the ward and stay in touch after leaving hospital, which could be harmful. Additionally, they highlighted the potential for young people to learn maladaptive behaviours, including self-harm, from older patients during their stay (Quote 5, Table 3).

Parents conveyed similar concerns about the influence of older patients, and their child being exposed to self-harm and suicidal behaviours on the ward, and potentially adopting these behaviours (Quote 6, Table 3). One parent said the adult ward was ‘horrible’ for her son, describing him as ‘quite young for his age and still wants to play with toys and things’. Young people relayed that they had felt frightened during their stay on the adult ward. One wanted to remain in the designated ‘Kids’ room’ where they had been kept at the start, and another said they were moved to a different facility because of concerns over their safety. Generally, young people did not feel comfortable mixing with the adults on the ward (Quote 7, Table 3), with one describing the adult patients as ‘intimidating’. One young person, who had witnessed a fellow patient’s suicide attempt, described it as ‘one of the worst experiences I’ve had’. The young person attributed their worsening mental health to this experience (Quote 8, Table 3).

#### Lack of education provision and age-appropriate activities

Young people often reported feeling bored and isolated when staying on an adult ward, because of the lack of age-appropriate activities and education provision, coupled with limited opportunities for social interactions (Quotes 9–11, Table 3). By contrast, parents and young people spoke positively about their experiences when young people were admitted to adolescent in-patient wards, because these wards provided structured environments, activities and schooling. MHPs highlighted that young people could push boundaries on the adult wards because there is little else for them to do. They acknowledged that their efforts to engage with young people were contingent on staffing levels and the resources available (Quote 12, Table 3).

#### Wards may be clinically or socially appropriate for a small subset of young people

Despite their strong views overall, a small number of MHPs felt that in rare cases, an adult ward might occasionally be more clinically or socially appropriate for young people with particularly adult needs. They therefore felt admission should not be a ‘never event’. They instead supported a nuanced approach to admissions, proposing that placements should be contingent on factors such as a young person’s age and presenting issues. Notably, an adult ward might be considered more appropriate for a young person who lives independently and in employment rather than education, or who may find the restrictions of a CAMHS ward challenging (Quotes 13 and 14, Table 3). They emphasised that most admissions tend to be male patients, approaching 18 years of age and presenting with psychosis and aggressive behaviour. The decision to admit these patients was often influenced by challenges in managing their behaviour in adolescent wards and the risk they posed to other young people (Quote 15, Table 3). Moreover, an adult mental health ward may provide the specialist skills and support considered necessary to meet the patient’s clinical needs.

### Theme 3: impact on services and provision of care

#### Level of care for young people on adult wards

Professionals reported that there is a lack of specific guidance for adult clinical teams to manage and treat young people. Challenges emerge as a result of staff not having the specialised knowledge or experience in working with adolescents (Quote 1, Table 4). In one example, staff found it particularly difficult to manage a young person whose level of functioning was significantly below their chronological age (Quote 2, Table 4). MHPs acknowledged that ‘training with regards to how to deal with younger people could be useful’.

Despite this, some MHPs believed that young people received a high level of care during their admissions. For instance, these types of admissions frequently trigger daily escalation calls, bringing together a multidisciplinary team, and services are mobilised quicker than usual. Receiving units provide a bespoke care package, and some adult MHPs felt this provided more rehabilitation than the average adolescent ward. They noted the difference between CAMHS and AMHS in terms of their ‘philosophy’ toward treatment and care (Quote 3, Table 4): AMHS was described as being less paternalistic and risk averse than CAMHS, and more focused on empowering individuals. For some presentations, encouraging young people to take responsibility for their behaviour was considered a beneficial aspect of care (Quote 4, Table 4).

Although parents and young people acknowledged the supportiveness of staff on the adult ward, they also noted a lack of expertise among staff in managing young patients (Quote 5, Table 4). Compared with CAMHS, there was notably less communication, which some parents found frustrating because they wanted to be kept informed. However, one parent expressed relief at the reduced contact with staff, as it provided them with much-needed respite. Additionally, parents and young people reported a lack of treatment and therapy during their stays, and felt there was more of an emphasis on patient safety, highlighting a contrast with their experiences in adolescent in-patient units (Quotes 6–9, Table 4).

#### Some safeguarding protocols are felt to be intrusive and restrictive

Because of concerns about the safety of a young person on an adult ward, there is often a focus on safeguarding measures. In some units, young people are admitted to a segregated part of the ward and the adult patients are vacated from communal areas when a young person is present. A standard practice also involves all young people being automatically placed on one-to-one observation, with two levels of proximity; for example, level 3 (within eyesight) or level 4 (within arm’s length). MHPs said that most young people find the high levels of observation to be intrusive, e.g. patients are monitored in the bathroom, potentially exacerbating feelings of paranoia in some patients. Although protocols are carefully explained to young people, many still found these procedures to be restrictive and isolating (Quotes 10 and 11, Table 4). MHPs expressed reservations about the universal use of one-to-one observation and felt that there should be an element of clinical judgement within these decisions, as there would be for any other patient.

#### Barriers and facilitators to collaborative working

Professionals discussed the barriers to collaborative work and how the current infrastructure, and the separation of AMHS and CAMHS, does not readily support the sharing of information across services or specialties. Once a young person is admitted to the adult ward, the involvement of their CAMHS consultant is initiated (Quote 12, Table 4); however, in some regions, CAMHS consultants are not on-call overnight, and therefore not available to provide timely information and advice to AMHS consultants. A key barrier to collaborative work arises from the divergent approaches toward patient risk and management between AMHS and CAMHS, resulting in potential disagreements, especially concerning medication and discharge timelines. Some AMHS clinicians felt an obligation to adhere to CAMHS management plans, even if they considered them not clinically beneficial for the patient under their care. It was acknowledged that joined-up working would require effective communication between services, as well as increasing resource and staffing levels (Quote 13, Table 4).

Noteworthy examples of successful collaboration tend to happen when community and CAMHS crisis teams visit the young person during their stay on the adult ward, facilitating a seamless continuity of care. Effective communication emerged as a crucial element for successful collaborative working, with some MHPs noting positive and effective working relationships between adult and adolescent clinical teams. However, there was a perceived need for better collaboration with social care to improve the processes for discharging young people (Quotes 14 and 15, Table 4).

### Theme 4: recommendations for service provision and policy

#### Integration of services

MHPs advocated for an integrated pathway in which both CAMHS and AMHS teams are involved in the decisions regarding treatment plans and discharge. Such an approach would ensure the comprehensive involvement of relevant teams in the young person’s care (Quote 1, Table 5). They reported that limited information is passed from CAMHS at admission, prompting some professionals to suggest a shared notes system that would facilitate information sharing between services.

Consultants discussed some complex cases and the challenging decisions about whether a young person should be admitted to an adult or adolescent ward. They provided some successful models of care e.g. some regions introduced joint assessment panels with tripartite funding (from the NHS, Social Care and Education) for more complex cases, such as those involving young people at risk of extended in-patient admissions. Furthermore, some regions have implemented a transitional service for 16- to 25-year-olds, jointly operated by CAMHS and AMHS, which has the potential of being rolled out nationally. Suggestions were made for CAMHS and AMHS psychiatrists to meet weekly for supervision and guidance, facilitating smoother transitions between services (Quote 2, Table 5).

Parents expressed the need for greater coordination between mental health services and social care. One parent described the services as being at odds with each other, leaving their daughter caught up in the middle (Quote 3, Table 5).

#### Increase community provision

A strongly held view across all participants was the potential to reduce adult ward admissions through earlier community-based support (Quotes 4–6, Table 5). MHPs advocated for the provision of early intervention support within educational settings, to address concerns raised by families regarding their child’s mental health. The lack of adequate community care and support was identified as a key factor leading young people and their families into a preventable crisis, resulting in in-patient services becoming the default option (Quote 7, Table 5). They argued the need for better-resourced community teams equipped to address problems early on, to prevent escalation and facilitate timely access to services. Emphasis was placed on the necessity for well-supported home treatment teams capable of intensive community-based intervention (Quotes 8 and 9, Table 5).

In one particular region, a new care model was implemented to expand home treatment teams, aiming to reduce lengths of stay and to manage young people with longstanding mental health difficulties more effectively at home. Suggestions were made for home treatment models that reflect the successful community eating disorders model, in which a health professional visits the young person several times a day, working intensively with the family (Quote 10, Table 5). The suggestion of non-hospital respite centres in the community could be beneficial by providing young people with access to support, as well as providing families with respite from managing the daily safety risks. One MHP said that placing conditions on this could help young people to take responsibility for risk and their recovery.

According to adult clinicians, enhancing community services would necessitate increased recruitment and retention of professionals, including nurses, psychologists, doctors and a comprehensive multidisciplinary team, coupled with investment in staff training (Quote 10, Table 5). MHPs underscored the importance of providing out-of-hours support for young people. They cited an example of a home-based treatment team that operates at weekends, to intervene and prevent young people from resorting to emergency services. However, the lack of an overnight service was identified as a potential gap in some regions, especially considering that night-time is when young people are more likely to experience a crisis (Quote 12, Table 5).

#### Recommendations for policy

MHPs called for the implementation of flexible processes that would facilitate appropriate placements, thus averting prolonged stays in emergency services. A few MHPs felt that the decision of whether to admit a young person to an adult ward could be made on a case-by-case assessment, as there are rare cases (e.g. older adolescents who live independently) for whom an adult ward might be more appropriate than an adolescent ward. In instances when an admission to an adult ward is deemed necessary, clear guidelines defining individual roles and specifying the responsible clinician are essential (Quote 13, Table 5). One MHP recommended the development of a policy incorporating a time limit for a young person’s stay on an adult mental health ward, such as a maximum duration of 72 hours, with consequences for breaching this limit to deter inappropriate admissions.

It was suggested that clinicians should have some discretion in deciding whether a patient aged <18 years requires one-to-one observation. Some emphasised the importance of affording greater weight to clinical judgement in determining whether high observations were required, cautioning against blanket policies that may prove rigid and restrictive. The decision-making process should be driven by an assessment of risk, rather than chronological age, with safeguarding considerations lending support to these decisions (Quote 14, Table 5).

MHPs recommended forming guidelines for the transition from adolescent to adult wards, similar to existing guidance on transition in the community. In particular, a policy addressing the discharge of young adults who have spent significant time in CAMHS was proposed, since they are particularly vulnerable. Further research and comprehensive guidance would determine the appropriateness of admissions for specific young individuals. Parents and young people highlighted the importance of supporting patients and families with transition from CAMHS to AHMS (Quote 15, Table 5).

## Discussion

### Main findings

This national study investigated the impact of young people’s admissions to adult wards, from the perspective of young people, parents/carers and MHPs who work in adult services. The findings show that such admissions often occur out of hours, at a time of crisis, when no suitable adolescent beds are available. Adult ward admissions are perceived primarily as a short-term safety measure in a critical situation, as opposed to providing any therapeutic intervention. However, some young people experienced prolonged stays arising from challenges in securing a CAMHS bed, during which there was limited therapy and treatment provided. The findings underline that adult mental health wards are generally perceived to be inappropriate for young people because of concerns about patients’ comfort and safeguarding, and young people not receiving education or age-appropriate care. Nevertheless, very occasionally, an adult facility may be deemed more clinically or socially appropriate, depending on the patient’s age and presentation. Recommendations for enhancing service provision include augmenting community services, better integration of adolescent and adult services and enabling flexible policies and processes.

### Strengths and limitations

This unique study provides new insights into the perspectives and experiences of patients, parents/carers and professionals regarding young people’s admissions to adult wards. A strength of the study is the relatively large sample size of MHPs from AMHS and representation from every NHS region in England, in addition to interviewing young people and parents/carers from three large regions in England. Advisory groups played a pivotal role, contributing to the refinement of research procedures and the analysis process.

There are several limitations to the research described in this paper. Although we were able to recruit MHPs from all regions in England, some consideration should be given to the transferability of the findings to other areas of the UK and other countries. Although the service provision is specific to the UK’s health service, participants’ views about these types of admissions may also be relevant to adolescent mental health services in many other countries. Our study only captured the views of MHPs working in AMHS, and did not include the perspectives of practitioners in CAMHS, who may hold differing views. Therefore, we must be cautious about making policy recommendations for adult ward admissions for young people aged <18 years based solely on the views of practitioners from adult services.

We acknowledge that the small sample size of young people and parents is a limitation of the study. Because of the differing sample sizes, the themes may be more reflective of MHPs’ perspectives than parents and young people. However, we felt it crucial to include young people and parent/carer perspectives. Recruiting in this setting was challenging, and further affected by the restrictions of the COVID-19 pandemic and ward closures, resulting in limited representation of these groups. Consequently, the views of participants, although valuable, may not fully encapsulate the diverse experiences and perspectives of all patients and families. It is important to acknowledge that those who chose not to participate may hold differing views and experiences. Therefore, further research with this group is needed.

As a guiding principle, we referred to the concept of information power, which suggests that the more relevant information a sample holds for the study, the fewer participants are needed.^25^ Despite the small sample size, the in-depth interviews with young people and parents yielded rich dialogue and detailed information about patient and carer experiences. Moreover, we believe it is essential to include participants with lived experience, to ensure their perspectives inform this research.

### Comparison with existing literature

This study provides qualitative evidence regarding the scope and nature of young people’s admissions to adult wards. Despite national policy specifying that young people be admitted to age-appropriate facilities,^13,14^ increased demand for in-patient care means that young people aged <18 years continue to be admitted to either out-of-area units or adult wards.^17^ Consistent with previous research, our findings show that admissions to adult wards typically occur in a crisis situation, and tend to involve young people who are at high risk and/or have been detained under the MHA.^15,17^ Such admissions relate to a lack of CAMHS in-patient provision, either because adolescent wards are full or unable to admit on an urgent basis,^7,8^ or because some have limited or no access to adolescent beds locally.^7,9^ The varied distribution of CAMHS in-patient facilities across the country may explain why professionals in the present study reported different experiences regarding the number of cases in their service. The urgency with which the referral happens can mean that patients and their families are not fully involved or informed, and are therefore unprepared for the reality of an adult ward environment.^20^

Similar to previous work, our findings highlight serious concerns about the safety and vulnerability of young people on adult wards, particularly patients aged 16 years or younger.^9,16,18^ A survey of UK psychiatrists reported several adverse experiences for young people, and found that children as young as 12 years had been admitted to adult wards while awaiting an adolescent bed.^16^ Similarly, in other studies, young people have reported negative experiences, such as feeling frightened and unsafe around older patients, as well as feeling isolated and bored because of a lack of education and age-appropriate activities on the adult ward.^18,20^ We also found that young people experienced a lack of therapeutic input on the ward. Previous research has also found that staff may lack the skills, expertise and confidence to manage young patients,^18^ which may adversely affect patient care. A concern is that negative experiences of mental healthcare could result in young people disengaging from services or seeking further help, once discharged.^26^ A risk that should not be underestimated given the delicate relationship between mental health services and young patients, especially at points of transition such as discharge.^27^

In a very small proportion of cases, admissions to adult wards may be considered clinically or socially appropriate. We found that clinicians did not like the use of blanket national policies that decide an adult ward is not appropriate,^13,14^ or the rule that every young person aged <18 years must be on a one-to-one observation regimen, but would prefer case-by-case consideration of what is best for the young person. A recent national surveillance study confirmed that most young people aged <18 years who were admitted to adult wards were aged 17 years,^17^ and older adolescents may prefer to be in adult mental health wards. Moreover, an adult ward may be preferable for the patient and their family because it is nearer to home. They may want to avoid being admitted to an adolescent ward that is out of area and would separate them from crucial support networks.^22^

### Implications for clinical practice and policy

The findings from this study aim to inform and improve the care of adolescent patients requiring in-patient care. Our findings align with current government policy that emphasises the importance of admitting young people to age-appropriate in-patient facilities.^13,14^ This study highlights that adult ward staff at all levels, including more senior staff, need appropriate support, training and protocols for the management of adolescents in their ward, as these types of admissions will continue to arise from time to time. Professional bodies may need to consider how the current provision of pre- and post-registration training could be improved. However, adult ward teams need flexibility with protocols, depending on the patient’s age and presentation. Our research suggests that there is a need for closer working and better integration between CAMHS and AMHS. This collaboration would facilitate the effective transfer of information, ensure that staff feel adequately supported and that young people receive age-appropriate care. Investing in community and crisis care services would be an effective strategy to help prevent need for hospital admission and reduce demand for in-patient resources.^28^

## Supporting information

Burn et al. supplementary material 1 Burn et al. supplementary material

Burn et al. supplementary material 2 Burn et al. supplementary material

Burn et al. supplementary material 3 Burn et al. supplementary material

Burn et al. supplementary material 4 Burn et al. supplementary material

Burn et al. supplementary material 5 Burn et al. supplementary material

## Data Availability

The qualitative data that support the findings of this study are available from the corresponding author upon reasonable request. The data are not publicly available as they contain information that could compromise the privacy of research participants.
