# Supplementary material for: Impact of young people’s admissions to adult mental health wards in England: national qualitative study
Source: BJPsych Open. 2025 Mar 17;11(2):e53. doi: 10.1192/bjo.2024.850 (PMC12001951; doi:10.1192/bjo.2024.850)
Supplement: Burn et al. supplementary material 2 — Burn et al. supplementary material [file S2056472424008500sup002.pdf]

Supplementary Materials 1: Young Person Interview Schedule

Topic & Timing	Discussion Point	Prompts
**Contextual background**<br><br>(5 mins)		• Contextual information about participant e.g. family, friends, work/study to get participant talking and to feel comfortable
**Circumstances leading to admission**<br><br>10 minutes	"Can you tell me, first of all, about your recent experience of being a patient at [insert name of hospital/ward]?"<br><br>What was it like at the beginning of admission?<br><br>How did it change?	• Events leading up to admission<br>• Process of referral and how it was initiated<br>• How did YP feel about being referred to unit<br>o Relieved, apprehensive, unnecessary<br>• To what extent did she/he feel consulted, involved in decisions about care<br>o Did YP feel she/he had a choice? Was this important?<br>• What were their expectations of the unit, before she/he was admitted?
**Experiences during admission**<br><br>10 minutes	"Can you tell me what happened, once you were admitted? What was it like being a patient at [insert name of hospital/ward]?"<br><br>"How was it being away from home?"	• Environment<br>o Other patients<br>o Staff<br>o Information, communication, support, trust<br><br>Treatment<br>o Type, effect, acceptability, Involvement in decisions<br><br>Length of stay<br>o Visits/support from family and friends<br>o any issues, difficulties experienced in visiting?<br><br>Did you find it helpful to be in the unit?<br>o In what way? What did you like most?<br>o Were there any things you did not like?<br><br>Do you think that being admitted to [insert name of hospital/ward] helped you get better?
**Type of unit specific**	"How did you feel about being admitted to a / distant adolescent unit / adult psychiatric ward?"	• Positive / negative experiences (e.g. homesick, stigma)<br>• How much input did you have in the decision to admit you to this unit?<br>• Was [insert name of hospital/ward] an appropriate place for someone of your age?<br>• Do you think [insert name of hospital/ward] was the best place for you at the time?<br>• Was there a better alternative?<br>• Did the fact that the admission was going to be out-of-area/in an adult ward influence your willingness to agree to it?<br>• What were the most difficult aspects of being in an out-of-area admission/on an adult ward?<br>• Was there any positive aspect to being in an out-of-area admission /on an adult ward?<br>• If your treating team recommended another admission in the future and you knew that it is going to be out-of-area/on an adult ward, would you agree to it?
**Comparisons with other admissions**<br><br>5 minutes	"Was this the first time you had been admitted as an inpatient?"	• If no – explore previous admissions, as above.<br><br>• Covid-19 experience

**Circumstances post admission**<br><br>5 minutes	"Since you came home after being in [insert name of hospital/ward], how have things been? Can you tell me how things are just now?"	• Health (any treatment; what does YP think of this; could it be improved/made more acceptable?)<br>• Home circumstances<br>• School<br>• Relationships<br>• Do you have any idea about what you want to do in future?<br>• Aspirations re health, job etc
**Reflection**<br><br>10 minutes	"Looking back, what do you think about the time you spent in [insert name of hospital/ward]"	• Helpful/unhelpful<br>• good/positive or bad or negative effects?<br><br>• anything else that helped you get better<br>Friends, family, support groups, activities etc : relative importance, compared to admission and professional help<br><br>• What do you think needs to happen to reduce admissions of young people to distant adolescent units / adult psychiatric wards (as appropriate)?<br><br>• Can you think of any ways that the experience of young people in your situation when you were admitted to [insert name of hospital/ward] could be improved:<br><br>• Finally, what advice would you give someone who was in your situation, just before you went to [insert name of hospital/ward]?
**Ending & Sum Up**<br><br>5 minutes	"Is there anything else you have to add to what we have been talking about? Anything that is important that we haven't covered already?"	• Thank them for their participation<br>• Explain timeline and output of the study and how access to results will be provided.
