# Supplementary material for: Impact of young people’s admissions to adult mental health wards in England: national qualitative study
Source: BJPsych Open. 2025 Mar 17;11(2):e53. doi: 10.1192/bjo.2024.850 (PMC12001951; doi:10.1192/bjo.2024.850)
Supplement: Burn et al. supplementary material 3 — Burn et al. supplementary material [file S2056472424008500sup003.pdf]

Supplementary Materials 2: Parent/Carer Interview Schedule

**Topic & Timing**	**Discussion Point**	**Prompts**
**Background**<br><br>5 minutes	*Can you tell me, first of all, about your recent experience of (Young person's) admission to XXXXXX?*	Seek to establish a detailed narrative account, including<br>Events leading up to admission<br>Process of referral and how it was initiated<br>How did YP and R/other family members feel about being referred to unit?<br>• Relieved, apprehensive, unnecessary, resisted etc<br>To what extent did R feel that the YP and family were consulted, involved in decisions about care?<br>• What is actual, appropriate and desired involvement of family in discussion and decisions about care?<br>• Did R feel that there was a choice of options for (YP) Was this important?<br>• What were R's expectations of the unit, before YP was admitted?
**Experience**<br><br>10 minutes	*Can you tell me what happened, once YP was admitted? What was it like for her being a patient at XXXXX?*	Experience of<br>• Environment<br>• Other patients<br>• Staff<br>• Information, communication, support, trust<br>• Treatment<br>• Type, effect, acceptability<br>• Involvement in decisions<br>• Length of stay<br>• Visits/support from family and friends<br>Did you find it easy to keep in touch with YP and provide support while she was on the unit?<br>• Frequency and duration of visits<br>• What happened during visits?<br>• Issues of privacy/environment<br>• Any issues, difficulties experienced in visiting<br>• Time and travel (distance)<br>• Need for time of work<br>• Problems with other commitments such as child care<br>• Any additional costs incurred?<br>Do you think YP found find it helpful to be in the unit?<br>In what way? What did think helped them most?<br>Were there any things you did not like/felt were not helpful?<br>Do you think that being admitted to xxxxxx helped YP get better?<br>Was xxxxxxxx an appropriate place for someone of her age?<br>Do you think xxxxxxxxxxxxx was the best place for her at the time?<br>Was there a better alternative?<br>How do you feel about your/family's involvement in YP's care while she was admitted to XXXXXXXX?<br>Information about what was happening and treatment plan<br>Access and communication with staff<br>Discussion and involvement in decision making
**Other experiences**<br><br>5 minutes	*Was this the first time YP had been admitted as an inpatient?*	If no – explore previous admissions, as above.

**Post admission**<br><br>10 minutes	*Since YP came home after being in XXXXXX, how have things been? Can you tell me how things are just now?*	Health (any treatment)<br>Home circumstances<br>School<br>Relationships<br>Do you have any idea about what YP will want to do in future?<br>Aspirations re health, job etc<br>Do you have any concerns about this?
**COVID-19**<br><br>5 minutes	*How was your experience during the COVID-19 pandemic*	TBC - Covid-19 experience<br><br>Difference with other admissions if relevant
**Reflection**<br><br>5 minutes	*Looking back, what do you think about the time YP spent in xxxxxxx*	Helpful/unhelpful<br><br>Do you think it has had any good/positive or bad or negative effects? (as appropriate)<br><br>Was there anything else that helped YP get better at this time? Friends, family, support groups, activities etc: relative importance, compared to admission and professional help<br><br>Can you think of any ways that the experience of young people in YP's situation when she was admitted to XXXXXX could be improved:<br><br>Finally, what advice would you give someone who was in your situation, just before YP went to XXXXXXX?
**Ending & Sum Up**<br><br>5 minutes	*"Is there anything else you have to add to what we have been talking about? Anything that is important that we haven't covered already?"*	
