# Supplementary material for: Impact of young people’s admissions to adult mental health wards in England: national qualitative study
Source: BJPsych Open. 2025 Mar 17;11(2):e53. doi: 10.1192/bjo.2024.850 (PMC12001951; doi:10.1192/bjo.2024.850)
Supplement: Burn et al. supplementary material 4 — Burn et al. supplementary material [file S2056472424008500sup004.pdf]

Topic & Timing	Discussion Point	Prompts
**Case Example background and circumstance**<br><br>10 minutes	*"Can you tell me, first of all, about a recent case involving referral of a young person to an inpatient adult unit"*	Seek to establish a detailed narrative account, including<br>Events leading up to referral<br>What factors indicated that inpatient admission was necessary?<br>were there any other options?<br>Process of referral and how it was initiated discussion with other health professionals, YP and family<br>How did the YP and other family members feel about being referred to unit?<br>Relieved, apprehensive, unnecessary, resisted etc<br>To what extent did you feel that the YP and family were consulted, involved in decisions about care?<br>Do you think YP found it helpful to be in the unit? In what way? What did think helped them most?<br>Do you think adult ward staff have the skills and experience necessary to care for YP under the age of 18?<br>Were there any negative aspects or consequences of the admission?<br>e.g. difficulties of YP's integration with adult patients and ward activities, safeguarding issues, impact on other patients, consequences for staff time and work load<br>How did you find communication and contact with other health professionals, including CAMHS, involved in YP's care?<br><br>How would you assess the outcome of the YP's admission?<br><br>Do you think (the ward) was an appropriate place for someone of YP's age?<br><br>If not, what would have been the best place?
**Impact of admission**<br><br>5 minutes	*"What are the consequences for YP of being admitted to an adult ward: negative and positive?"*	e.g. re experience of being an inpatient, contact with family, contact with familiar services and health professionals, ease of reintegration into family, school, community, etc<br>Do inpatient admissions pose any issues regarding continuity of care and the YP's engagement with CAMHS following discharge home?<br>Do you think that some YP might prefer admission to an adult, rather than a children's unit, or is this always inappropriate or less than ideal? (Explore)

**Experience and Perceptions**<br><br>10 minutes	YP was admitted to an adult ward. How often does this happen?	Under what circumstances (choice, necessity etc)<br><br>What is your view about young people being admitted to adult wards?<br><br>How regularly does this happen?<br><br>Who refers these patients?<br><br>Do you receive appropriate information in the documentation?<br><br>Do you have a protocol for managing young people on adult wards? If yes, useful or not? If no, how do you plan?<br><br>What involvement do you have in planning and managing the process of these admissions?<br><br>To what extent do you feel that young people (aged 13-17 years) can be properly cared for on adult wards?<br><br>How confident do you feel in engaging with these young people?<br><br>What specific of issues do you experience when a young person is admitted to your adult ward?<br><br>How does admitting a young person to an adult ward impact on the treatment that can be offered?<br><br>How do you feel admission to an adult ward affects the patient and family experience?<br><br>How are CAMHS consultants / GPs involved in joint working in such cases? Barriers? How could this be improved?<br><br>What involvement do you have in planning continuity of care / post discharge care? Who else is involved in this? How easily is this achieved? How could it be improved?<br><br>Where referrals are not accepted, what are the reasons? Is an alternative offered?<br><br>What do you think needs to happen to reduce admissions of young people to distant adolescent units / adult psychiatric wards (as appropriate)?<br><br>What is the frequency of having a young person under 18 admitted to your ward?<br><br>What are the most difficult aspects of having a young person under 18 on the ward?

		Was there anything positive in the experience of having young person under 18 on the ward?<br><br>Did you find your Child and Adolescent Psychiatry colleagues helpful or unhelpful? In what ways were they helpful or unhelpful?<br><br>What are your thoughts about the effect of the admission to an adult ward on the young person and their family?
**Comparisons with other admissions**<br><br>5 minutes	*"How does your experience of cases such as these differ during the COVID-19 pandemic*	
**Reflection**<br><br>5 minutes	*"Do you think it should be possible for people in YP's situation to avoid inpatient admission, and be treated in the community?"*	what would be required to enable this to happen?
**Policy/Guidelines**<br><br>10 minutes	*"How do current practice guidelines and policy drivers inform your care of young people like XX(YP)?"*	Are there any changes in current policy/clinical guidelines that you feel would improve current care of YP in psychiatric services?<br><br>Are there any changes you would like to see in the organisation and resources available to you in managing the care of YP with severe mental health problems?
**Ending & Sum Up**<br><br>5 minutes	*"Is there anything else you have to add to what we have been talking about? Anything that is important that we haven't covered already?"*	

Thank you very much for your help with our research and for taking part in this interview.

Explain timeline and output of the study and how access to results will be provided.
