# Supplementary material for: Impact of young people’s admissions to adult mental health wards in England: national qualitative study
Source: BJPsych Open. 2025 Mar 17;11(2):e53. doi: 10.1192/bjo.2024.850 (PMC12001951; doi:10.1192/bjo.2024.850)
Supplement: Burn et al. supplementary material 5 — Burn et al. supplementary material [file S2056472424008500sup005.pdf]

Supplementary Materials 4: Commissioner Interview Schedule

Topic & Timing	Discussion Point	Prompts
**Service provision**<br><br>5 minutes	*"Can you give me an outline, first of all, of how specialist psychiatric services for young people (aged 13-17) are configured in xxx area?"*	Community/CAMHS, inpatient
**Frequency and Process**<br><br>5 minutes	*"To what extent to you think there is an issue with young people (aged 13-17 years) child being admitted to distant adolescent units / adult psychiatric wards? "*	Do you know how many YP within your commissioning area are admitted to 1. local specialist in patient units 2. specialist units at distance from home and 3. local adult wards?<br><br>What factors necessitate inpatient admission?<br>• How is the process of referral negotiated between different health professionals and services?<br>• What is your role in organising admissions and identifying suitable placements?<br><br>Once a YP has been admitted to an adult ward or distant unit, do they remain there until discharge, or would the attempt be made to find an alternative, for example if a more appropriate or local placement became available?<br>• What is the current policy about this?<br>• Is it possible to follow it?
**Impact of admission - individual**<br><br>10 minutes	*"What are the consequences for YP of being admitted to an adult ward: negative and positive?"*	*"Do you have any knowledge of how YP and their families feel about inpatient admissions and specifically to adult units or specialist adolescent units at some distance from home?"*<br><br>What do you see as the challenges of such admissions for<br>• YP and their families<br>• the professionals and units providing care?<br><br>How would you assess the outcome of YPs' admission to adult wards or units far away from home?<br><br>• e.g. re experience of being an inpatient, recovery, contact with family, contact with familiar services and health professionals, ease of reintegration into family, school, community, etc<br><br>• Do you think that some YP might prefer admission to an adult, rather than a children's unit, or is this always inappropriate or less than ideal? (Explore)
**Service provision impact**<br><br>10 minutes	*"Do (different kinds of) inpatient admissions pose any issues regarding continuity of care and the YP's engagement with CAMHS following discharge home?"*	From the commissioning perspective, what do you consider to be the challenges in providing appropriate care for YP affected by severe mental health problems?<br>What things work well?
**Comparisons**<br><br>5 minutes	*"How does your experience of cases such as these differ during the COVID-19 pandemic*	

**Reflection**<br><br>5 minutes	*"Are there any changes you would like to see in the organisation and resources available to you in managing the care of YP with severe mental health problems?*	what would be required to enable this to happen?<br><br>What do you think are the main disadvantages of these admissions over local adolescent units? Any advantages?<br><br>Any suggestions on changes that could be made to reduce admissions of young people to distant adolescent units / adult psychiatric wards?<br><br>Any suggestions on changes that could be made to improve the process / experience associated with admissions to distant adolescent units / adult psychiatric wards?
**Policy/Guidelines**<br><br>5 minutes	*"How do current practice guidelines and policy drivers inform your care of young people like XX(YP)?"*	Do you think it should be possible for people in YP's situation to avoid inpatient admission, and be treated in the community?<br>○ what would be required to enable this to happen?<br><br>Are there any changes in current policy/clinical guidelines that you feel would improve current care of YP in psychiatric services?<br><br>Are there any changes you would like to see in the organisation and resources available to you in managing the care of YP with severe mental health problems?<br><br>• Do you think it should be possible for YP with severe mental health problems to avoid inpatient admission, and be treated in the community? Or is admission the most appropriate option in some cases?
**Ending & Sum Up**<br><br>5 minutes	*"Is there anything else you have to add to what we have been talking about? Anything that is important that we haven't covered already?"*	
